# The predictive value of liver tests for the presence of liver metastases

**DOI:** 10.2217/hep-2023-0003

**Published:** 2023-10-12

**Authors:** Alexandra V Kimchy, Harjit Singh, Esha Parikh, Jessica Rosenberg, Kavya Sanghavi, James H Lewis

**Affiliations:** 1 Department of Internal Medicine, MedStar Georgetown University Hospital, Washington, DC 20007, USA; 2 Department of Gastroenterology, MedStar Georgetown University Hospital, Washington, DC 20007, USA; 3 MedStar Health Research Institute, Hyattsville, MD 20782, USA

**Keywords:** breast neoplasms, colonic neoplasms, liver biochemical tests, liver metastases, lung neoplasms, malignant melanoma

## Abstract

**Aim:**

To analyze the predictive value of biochemical liver tests in patients with malignant melanoma, breast, colorectal or lung cancers at the time of diagnosis of liver metastases.

**Methods:**

A retrospective review of patients with the above-mentioned solid tumors at MedStar Georgetown University Hospital from 2016–2020.

**Results:**

The highest optimal cutoff according to sensitivity and specificity for the presence of liver metastases was for AST ≥1.5 × ULN for melanoma, lung, and breast cancers and ≥2 × ULN for colorectal cancer, ALT ≥1.25 × ULN for melanoma, breast and colorectal cancers and ≥1.5 × ULN for lung cancer, and ALP ≥1.5 × ULN for melanoma, breast and colorectal cancers.

**Conclusion:**

Using thresholds of liver enzymes above the ULN may improve the diagnostic accuracy for the presence of liver metastases.

## Tweetable abstract

We determined the predictive value of biochemical liver tests in patients with malignant melanoma, breast, colorectal or lung cancers at the time of diagnosis of liver metastases. Read it here now: #openaccess #livercancer.

The liver is a frequent location of metastatic disease having a dual blood supply facilitating spread from nearby sites as well as serving as a selective target for more distant tumors [1,2]. In patients with cancer, as many as 50% will have metastases to the liver on initial presentation or with later progression of their primary malignancy [3,4]. Surveillance, Epidemiology and End Results registry data have shown that liver metastases are detected in nearly 5% of patients at the time of the primary cancer diagnosis, although there is variation across malignancies [5]. For instance, liver metastases are discovered upon diagnosis of colorectal cancer in approximately half of cases of colorectal liver metastases [6]. Some of the most common tumors metastasizing to the liver include colorectal cancer, breast cancer, lung cancer and melanoma with colorectal being the predominant primary site [3].

Metastases to the liver generally portend unfavorable outcomes with a 5-year survival rate approaching zero if left untreated [7]. Patients with colorectal liver metastases have significantly reduced survival rates compared with those without liver metastases [8]. Furthermore, patients with colorectal metastases have a low life expectancy without treatment ranging from 5 to 9 months; therefore, timely diagnosis and treatment of metastases may improve patient prognosis [9,10]. In metastatic breast cancer, approximately 50% of patients will develop liver involvement during the course of their disease [11]. Median survival time is significantly reduced in breast cancer patients with liver metastases compared with those without liver metastases [12]. In one study, the median time from liver metastases to end of life was only 11 months [13]. The overall survival reported for lung cancer liver metastases is low with an associated 1-year survival of 19% for small cell lung cancer and median survival of 4 months for non-small cell lung cancer [14,15]. In patients with advanced stage melanoma, visceral metastases are associated with a poor prognosis having median survivals of 2–7 months in cases of hepatic involvement [16].

Early detection of liver metastases is essential for timely intervention with the currently available treatment modalities, including resection, ablation, and systemic therapies [3]. Although novel targeted therapeutic agents have demonstrated efficacy in managing metastatic disease, resection still holds the greatest potential for a cure [17]. Surgical resection can only be used for liver metastases that are within defined criteria; however, indications for resection have expanded over the years due to the long-term survival benefit and low mortality following hepatectomy [18]. Furthermore, the use of systemic therapies in combination with advanced surgical and ablative techniques may allow for more patients to become eligible for resection if metastases are diagnosed early in the disease course [16,19].

Establishing a clinical diagnosis of early liver metastases is challenging as many patients lack typical signs and symptoms of liver disease [3]. For instance, while nearly 77% of patients with melanoma have liver metastases on autopsy, only 10–20% present with clinically apparent disease [20]. Metastases to the liver are frequently detected incidentally or during surveillance of a primary malignancy using imaging techniques such as ultrasound, computed tomography (CT), MRI and PET [3,7]. Liver biopsy is not required to establish a diagnosis of metastasis but can be useful in cases where the primary tumor site is unknown and histologic confirmation is needed [3]. In addition, tumor markers such as CA 19-9, chromogranin A, CA 15-3 and CA-125 when elevated may suggest the presence of systemic metastases needing further evaluation [21]. However, they are more often specific to the primary cancer and not necessarily the site of metastasis [21]. As early detection is critical to patient prognosis, there is a need for rapid and cost-effective screening methods for liver metastases to direct further assessment [8].

Although routinely performed as part of the workup, the utility and predictive value of biochemical liver tests in the detection of liver metastases from common primary malignancies localizing to the liver remains unclear [8,13,22–27]. The literature evaluating the predictive ability of liver parameters for liver metastases has been inconsistent. In contrast to older studies, the results from more recent studies in patients with breast and colorectal cancers suggest liver enzyme elevations have utility in the detection of liver metastases [8,13,22,23,25]. For instance, median aspartate aminotransferase (AST), alanine aminotransferase (ALT), and alkaline phosphatase (ALP) were shown to be significantly higher in patients with breast liver metastases compared with those without liver metastases [13]. In one study of patients with colorectal cancer, median AST and ALT were significantly higher in patients with liver metastases than in those without liver metastases [8]. Another investigation in patients with colorectal cancer demonstrated that 77% of patients with liver metastases had elevated ALP above normal while only 33% of patients without liver metastases had elevations above normal and the difference was significant between groups [23]. The aim of the present study was to analyze biochemical liver tests in patients with liver metastases from melanoma, breast, colorectal or lung cancers at the time of diagnosis of metastases and determine their predictive values.

## Patients & methods

This case-control study received approval from the MedStar Health-Georgetown University Institutional Review Board. We retrospectively reviewed patient encounters from MedStar Georgetown University Hospital, an academic tertiary care and National Cancer Institute-designated Comprehensive Cancer Center, located in the District of Columbia from January 2016 through December 2020. The International Classification of Diseases, Ninth and Tenth Revisions were used to identify patients with primary cutaneous melanoma (ICD-9-CM 172 and ICD-10-CM C43), colorectal cancer (ICD-9-CM 153 and ICD-10-CM C18), breast cancer (ICD-9-CM 174 and ICD-10-CM C50), and lung cancer (ICD-9-CM 162 and ICD-10-CM C33, C34). The diagnosis of each cancer type was confirmed with tissue biopsy of the primary site documented in the electronic medical record (EMR) and patients without a biopsy confirmed diagnosis were excluded. Adult patients (>18 years) with liver metastases from each primary malignancy were identified using the diagnostic codes ICD-9-CM 197.7 and ICD-10-CM C78.7. The diagnosis of liver metastases was confirmed by CT, MRI, PET-CT and/or liver biopsy results documented in the EMR. Patients with a history of liver disease reported in the EMR and/or a diagnostic code for cirrhosis (ICD-9-CM 571.2, 571.5 and ICD-10-CM K7460, 7469, 7030, 7031) were also excluded to avoid confusion with abnormal liver tests at baseline. The control group consisted of patients who had a diagnosis of one of the four primary cancer types and were without demonstrable liver metastases or a prior/current history of chronic liver disease. These patients were propensity-matched in a 1 to 1 ratio to patients with liver metastases according to primary cancer type, age, and gender.

Patient demographics, symptoms, diagnoses, cancer treatments, and laboratory data were obtained from the documented encounter at the time of diagnosis of liver metastases with imaging and/or biopsy. The diagnosis of bone metastases was determined by CT, MRI, PET-CT and/or bone biopsy results from the EMR confirming the presence of metastases. The normal ranges for liver tests analyzed in our hospital laboratory were as follows: AST 0–33 U/L, ALT 10–49 U/L, ALP 46–117 U/L and total bilirubin (TB) 0.3–1.2 mg/dl.

We summarized the distribution of categorical and continuous variables using frequencies and percentages, and medians with interquartile ranges, respectively. Differences in medians were compared using the Kruskal Wallis or Wilcoxon rank-sum test as appropriate. Chi-square analyses were used to calculate differences in categorical variables. The sensitivity (SE), specificity (SP), positive predictive value (PPV), and negative predictive values (NPV) for abnormal values of liver tests in relation to liver metastasis were calculated using 2 × 2 contingency tables, along with the associated area under the curve (AUC). Statistical significance level was set at 0.05.

## Results

### Patient characteristics

#### Lung cancer

Of the 966 patients with lung cancer, 80 patients (8.3%) had a diagnosis of liver metastases confirmed on liver imaging and/or biopsy. Patients with and without liver metastases did not significantly differ in age, body mass index (BMI), or gender. Bone metastases were present in 54% of patients with liver metastases compared with 35% of patients without liver metastases (p = 0.02). There was a difference in prior or current cancer therapies received between patients with and without liver metastases (p = 0.047); however, the majority of patients with liver metastases had not yet received treatment of their primary malignancy. Most patients with liver metastases (81%) presented without symptoms indicative of liver disease (Table 1).

**Table 1.** Patient characteristics and biochemical liver test abnormalities in patients with and without lung cancer liver metastases.

Patient characteristics	Patients with liver metastases (n = 80)	Matched controls (n = 69)	p-value
Age (years)	65 (58, 73)	70 (60, 73)	0.235
BMI	23.72 (20.22, 28.48)	25.47 (21.73, 28.92)	0.144
Gender:			0.930
– Male	40 (50.00%)	34 (49.30%)	
– Female	40 (50.00%)	35 (50.70%)	
Race and ethnicity			0.130
– White	37 (46.30%)	43 (62.30%)	
– Black	28 (35.00%)	21 (30.40%)	
– Latina	1 (1.30%)	1 (1.40%)	
– Asian	11 (13.80%)	2 (2.90%)	
– Other	3 (3.80%)	2 (2.90%)	
Bone metastases	43 (53.80%)	24 (34.80%)	0.020^†^
Prior or current cancer therapy:			0.047^†^
– None	43 (53.80%)	24 (34.80%)	
– Chemotherapy	17 (21.30%)	13 (18.80%)	
– Target or immunotherapy	6 (7.50%)	9 (13.00%)	
– Chemo and targeted or immunotherapy	14 (17.50%)	23 (33.30%)	
Presenting symptom of liver metastases:			
– None	65 (81.25%)		
– Abdominal pain	11 (13.75%)		
– Weight loss	4 (5.00%)		
– Encephalopathy	3 (3.75%)		
– Jaundice	1 (1.25%)		
– Scleral icterus	1 (1.25%)		
AST	34.00 (20.00, 61.50)	20.00 (15.00, 26.00)	<0.001^†^
ALT	32.50 (18.50, 54.50)	21.00 (15.00, 27.00)	<0.001^†^
ALP	122.50 (89.00, 235.50)	89.00 (75.00, 112.00)	<0.001^†^
TB	0.50 (0.30, 0.90)	0.40 (0.30, 0.60)	0.036^†^
Elevation above normal range:			
– AST	41 (51.20%)	9 (13.00%)	<0.001^†^
– ALT	24 (30.00%)	6 (8.70%)	0.001^†^
– ALP	44 (55.00%)	16 (23.20%)	<0.001^†^
– TB	8 (10.00%)	1 (1.40%)	0.029^†^
AST × ULN:			0.284
– >1 to <1.25	8 (19.50%)	4 (50.00%)	
– ≥1.25 to <1.5	3 (7.30%)	1 (12.50%)	
– ≥1.5 to <2	12 (29.30%)	2 (25.00%)	
– ≥2 to <3	7 (17.10%)	1 (12.50%)	
– ≥3	11 (26.80%)	0 (0.00%)	
ALT × ULN:			0.487
– >1 to <1.25	6 (25.00%)	3 (50.00%)	
– ≥1.25 to <1.5	6 (25.00%)	2 (33.30%)	
– ≥1.5 to <2	3 (12.50%)	1 (16.70%)	
– ≥2 to <3	6 (25.00%)	0 (0.00%)	
– ≥3	3 (12.50%)	0 (0.00%)	
ALP × ULN:			0.145
– >1 to <1.25	10 (23.30%)	5 (33.30%)	
– ≥1.25 to <1.5	10 (23.30%)	5 (33.30%)	
– ≥1.5 to <2	4 (9.30%)	3 (20.00%)	
– ≥2 to <3	5 (11.60%)	2 (13.30%)	
– ≥3	14 (32.60%)	0 (0.00%)	

† Statistically significant (p ≤ 0.05).

Statistics presented as median (P25, P75) or frequency (%).

Elevation above normal range defined as AST >33, ALT >49, ALP >117, TB >1.2; Liver tests × ULN, percentage of patients within range of elevation listed out of total number of patients with elevated liver tests.

ULN: Upper limit of normal.

#### Breast cancer

Of the 859 patients with breast cancer, 50 patients (5.8%) had a diagnosis of liver metastases confirmed on liver imaging and/or biopsy. Patients with liver metastases had a higher median age than those without liver metastases (59 years vs 52 years; p = 0.025). Most patients with breast cancer were female and overweight with a median BMI of 29, which did not differ according to the presence or absence of liver metastases. Bone metastases were more often present in patients with liver metastases than in those without liver metastases (76% vs 17%; p < 0.001). There were no significant differences in prior or current cancer therapy types between groups. The majority of patients with liver metastases (78%) presented without symptoms indicative of liver disease (Table 2).

**Table 2.** Patient characteristics and biochemical liver test abnormalities in patients with and without breast cancer liver metastasis.

Patient characteristics	Patients with liver metastasis (n = 50)	Matched controls (n = 58)	p-value
Age (years)	59 (51, 66)	52 (47, 64)	0.025^†^
BMI	28.63 (23.31, 36.11)	28.51 (24.22, 32.15)	0.781
Gender:			0.279
– Male	1 (2.00%)	0 (0.00%)	
– Female	49 (98.00%)	58 (100.00%)	
Race and ethnicity:			0.005^†^
– White	8 (16.00%)	25 (43.10%)	
– Black	37 (74.00%)	24 (41.40%)	
– Asian	1 (2.00%)	4 (6.90%)	
– Other	4 (8.00%)	5 (8.60%)	
Bone metastases	38 (76.00%)	10 (17.20%)	<0.001^†^
Prior or current cancer therapy:			0.316
– None	4 (8.00%)	13 (22.41%)	
– Chemotherapy	12 (24.00%)	10 (17.24%)	
– Hormone therapy	8 (16.00%)	5 (8.62%)	
– Chemo and hormone therapy	13 (26.00%)	11 (18.97%)	
– Chemo & targeted or immunotherapy	3 (6.00%)	7 (12.07%)	
– Hormone and targeted or immunotherapy	1 (2.00%)	1 (1.72%)	
– Chemo, hormone and targeted or immunotherapy	9 (18.00%)	11 (18.97%)	
Presenting symptom of liver metastases:			
– None	39 (78.00%)		
– Weight loss	2 (4.00%)		
– Ascites	1 (2.00%)		
– Abdominal pain	8 (16.00%)		
– Other	0 (0.00%)		
AST	40.50 (25.00, 106.00)	19.00 (15.00, 26.00)	<0.001^†^
ALT	30.00 (22.00, 50.00)	21.00 (16.00, 30.00)	<0.001^†^
ALP	122.50 (95.00, 211.00)	77.50 (59.00, 113.00)	<0.001^†^
TB	0.40 (0.30, 0.80)	0.40 (0.30, 0.50)	0.022^†^
Elevation above normal range:			
– AST	30 (60.00%)	8 (13.80%)	<0.001^†^
– ALT	13 (26.00%)	3 (5.20%)	0.002^†^
– ALP	28 (56.00%)	13 (22.40%)	<0.001^†^
– TB	7 (14.00%)	2 (3.45%)	0.048^†^
AST × ULN:			0.03^†^
– >1 to <1.25	5 (16.70%)	4 (50.00%)	
– ≥1.25 to <1.5	2 (6.70%)	2 (25.00%)	
– ≥1.5 to <2	6 (20.00%)	1 (12.50%)	
– ≥2 to <3	4 (13.30%)	1 (12.50%)	
– ≥3	13 (43.30%)	0 (0.00%)	
ALT × ULN:			0.186
– >1 to <1.25	3 (23.10%)	3 (100.00%)	
– ≥1.25 to <1.5	3 (23.10%)	0 (0.00%)	
– ≥1.5 to <2	2 (15.40%)	0 (0.00%)	
– ≥2 to <3	0 (0.00%)	0 (0.00%)	
– ≥3	5 (38.50%)	0 (0.00%)	
ALP × ULN:			0.136
– >1 to <1.25	7 (25.00%)	6 (46.20%)	
– ≥1.25 to <1.5	3 (10.70%)	4 (30.80%)	
– ≥1.5 to <2	6 (21.40%)	2 (15.40%)	
– ≥2 to <3	6 (21.40%)	1 (7.70%)	
– ≥3	6 (21.40%)	0 (0.00%)	

† Statistically significant (p ≤ 0.05).

Statistics presented as median (P25, P75) or frequency (%).

Elevation above normal range defined as AST >33, ALT >49, ALP >117, TB >1.2; Liver tests × ULN, percentage of patients within range of elevation listed out of total number of patients with elevated liver tests.

ULN: Upper limit of normal.

#### Colorectal cancer

Of the 499 patients with colorectal cancer, 68 patients (13.6%) had a diagnosis of liver metastases confirmed with liver imaging and/or biopsy. Patients with liver metastases had a lower median age than those without liver metastases (61 yrs. vs 69 yrs.; p = 0.021). Median BMI and gender were not significantly different between groups. Bone metastases were present in few patients with colorectal cancer and the rates did not significantly differ among patients with and without liver metastases (7% vs 2%). There were no significant differences in prior or current cancer therapy types between groups. The most common presenting symptoms upon diagnosis of liver metastases were abdominal pain and weight loss (49% and 34%); however, symptoms indicative of liver disease were absent in 28% of patients (Table 3).

**Table 3.** Patient characteristics and biochemical liver test abnormalities in patients with and without colorectal cancer liver metastases.

Patient characteristics	Patients with liver metastases (n = 68)	Matched controls (n = 54)	p-value
Age (years)	61 (52, 70)	69 (57, 75)	0.021^†^
BMI	26.37 (22.71, 30.64)	26.31 (22.83, 30.97)	0.836
Gender:			0.203
– Male	35 (51.50%)	34 (63.00%)	
– Female	33 (48.50%)	20 (37.00%)	
Race and ethnicity:			0.564
– White	34 (50.00%)	25 (46.30%)	
– Black	25 (36.80%)	18 (33.30%)	
– Latina	1 (1.50%)	2 (3.70%)	
– Asian	2 (2.90%)	5 (9.30%)	
– Other	6 (8.80%)	4 (7.40%)	
Bone metastases	5 (7.40%)	1 (1.90%)	0.163
Prior or current cancer therapy:			0.475
– None	46 (67.60%)	38 (70.40%)	
– Chemotherapy	13 (19.10%)	10 (18.50%)	
– Target or immunotherapy	0 (0.00%)	1 (1.90%)	
– Chemo and hormone therapy	0 (0.00%)	1 (1.90%)	
– Chemo and targeted or immunotherapy	9 (13.20%)	4 (7.40%)	
Presenting symptom of liver metastases:			
– Abdominal pain	33 (48.53%)		
– Weight loss	23 (33.82%)		
– None	19 (27.94%)		
– Hematochezia	12 (17.65%)		
– Nausea and vomiting	7 (10.29%)		
– Jaundice	2 (2.94%)		
– Melena	1 (1.47%)		
AST	36.00 (20.50, 88.00)	20.50 (14.00, 30.00)	<0.001^†^
ALT	26.00 (19.00, 55.50)	23.50 (13.00, 34.00)	0.018^†^
ALP	135.00 (87.50, 285.50)	93.50 (72.00, 115.00)	<0.001^†^
TB	0.50 (0.40, 0.95)	0.40 (0.30, 0.60)	0.062
Elevation above normal range:			
– AST	35 (51.50%)	12 (22.20%)	0.001^†^
– ALT	20 (29.40%)	3 (5.60%)	0.001^†^
– ALP	38 (55.90%)	13 (24.10%)	<0.001^†^
– TB	10 (14.70%)	2 (3.70%)	0.043^†^
AST × ULN:			0.039^†^
– >1 to <1.25	3 (8.60%)	4 (40.00%)	
– ≥1.25 to <1.5	4 (11.40%)	2 (20.00%)	
– ≥1.5 to <2	7 (20.00%)	3 (30.00%)	
– ≥2 to <3	8 (22.90%)	1 (10.00%)	
– ≥3	13 (37.10%)	0 (0.00%)	
ALT × ULN:			0.531
– >1 to <1.25	3 (15.00%)	1 (33.30%)	
– ≥1.25 to <1.5	5 (25.00%)	0 (0.00%)	
– ≥1.5 to <2	5 (25.00%)	1 (33.30%)	
– ≥2 to <3	2 (10.00%)	1 (33.30%)	
– ≥3	5 (25.00%)	0 (0.00%)	
ALP × ULN:			0.001^†^
– >1 to <1.25	4 (10.50%)	7 (53.80%)	
– ≥1.25 to <1.5	4 (10.50%)	4 (30.80%)	
– ≥1.5 to <2	7 (18.40%)	1 (7.70%)	
– ≥2 to <3	12 (31.60%)	1 (7.70%)	
– ≥3	11 (28.90%)	0 (0.00%)	

† Statistically significant (p ≤ 0.05).

Statistics presented as median (P25, P75) or frequency (%).

Elevation above normal range defined as AST >33, ALT >49, ALP >117, TB >1.2; Liver tests × ULN, percentage of patients within range of elevation listed out of total number of patients with elevated liver tests.

ULN: Upper limit of normal.

#### Melanoma

Of the 130 patients with melanoma, 17 patients (13.1%) had a diagnosis of liver metastases confirmed on liver imaging and/or biopsy. Patients with and without liver metastases did not significantly differ in age, BMI, or gender. Bone metastases were present in 35% of patients with liver metastases and none of those without liver metastases (p = 0.009). There was no significant difference in prior or current cancer therapy types between groups. Although 47% patients with liver metastases did not present with symptoms indicative of liver disease, the most common symptom reported upon diagnosis of metastases was abdominal pain in 29% of patients (Table 4).

**Table 4.** Patient characteristics and biochemical liver test abnormalities in patients with and without melanoma liver metastases.

Patient characteristics	Patients with liver metastases (n = 17)	Matched controls (n = 16)	p-value
Age (years)	62 (56, 71)	62 (48, 71)	0.769
BMI	27.48 (23.89, 30.45)	26.18 (24.96, 33.11)	0.986
Gender:			0.353
– Male	9 (52.90%)	11 (68.80%)	
– Female	8 (47.10%)	5 (31.30%)	
Race and ethnicity:			0.374
– White	15 (88.20%)	13 (81.30%)	
– Black	0 (0.00%)	2 (12.50%)	
– Asian	1 (5.90%)	1 (6.30%)	
– Other	1 (5.90%)	0 (0.00%)	
Bone metastases	6 (35.30%)	0 (0.00%)	0.009^†^
Prior or current cancer therapy:			0.504
– None	6 (35.29%)	4 (25.00%)	
– Chemotherapy	0 (0.00%)	0 (0.00%)	
– Hormone therapy	0 (0.00%)	0 (0.00%)	
– Targeted or immunotherapy	11 (64.71%)	11 (68.75%)	
– Hormone and targeted or immunotherapy	0 (0.00%)	1 (6.25%)	
Presenting symptom of liver metastases:			
– None	8 (47.10%)		
– Jaundice	1 (5.90%)		
– Ascites	1 (5.90%)		
– Encephalopathy	2 (11.80%)		
– Abdominal pain	5 (29.40%)		
– Other	0 (0.00%)		
AST	46.00 (33.00, 82.00)	27.50 (23.00, 32.50)	0.006^†^
ALT	49.00 (29.00, 86.00)	33.00 (23.50, 37.50)	0.194
ALP	161.00 (116.00, 311.00)	95.50 (70.50, 105.00)	<0.001^†^
TB	0.60 (0.30, 1.10)	0.60 (0.35, 0.80)	0.865
Elevation above normal range:			
– AST	12 (70.60%)	3 (18.80%)	0.003^†^
– ALT	8 (47.10%)	2 (12.50%)	0.031^†^
– ALP	12 (70.60%)	3 (18.80%)	0.003^†^
– TB	4 (23.53%)	0 (0.00%)	0.038^†^
AST × ULN:			0.525
– >1 to <1.25	2 (16.70%)	2 (66.70%)	
– ≥1.25 to <1.5	2 (16.70%)	1 (33.30%)	
– ≥1.5 to <2	1 (8.30%)	0 (0.00%)	
– ≥2 to <3	3 (25.00%)	0 (0.00%)	
– ≥3	4 (33.30%)	0 (0.00%)	
ALT × ULN:			0.782
– >1 to <1.25	3 (33.30%)	1 (50.00%)	
– ≥1.25 to <1.5	1 (11.10%)	0 (0.00%)	
– ≥1.5 to <2	1 (11.10%)	1 (50.00%)	
– ≥2 to <3	1 (11.10%)	0 (0.00%)	
– ≥3	3 (33.30%)	0 (0.00%)	
ALP × ULN:			0.437
– >1 to <1.25	2 (16.70%)	2 (66.70%)	
– ≥1.25 to <1.5	2 (16.70%)	1 (33.30%)	
– ≥1.5 to <2	2 (16.70%)	0 (0.00%)	
– ≥2 to <3	2 (16.70%)	0 (0.00%)	
– ≥3	4 (33.30%)	0 (0.00%)	

† Statistically significant (p ≤ 0.05).

Statistics presented as median (P25, P75) or frequency (%).

Elevation above normal range defined as AST >33, ALT >49, ALP >117, TB >1.2; Liver test × ULN, percentage of patients with elevations within range listed out of total number of patients with elevated liver tests.

ULN: Upper limit of normal.

### Comparison of biochemical liver tests

#### Lung cancer

Median AST, ALT, ALP, and TB were slightly (but significantly) higher in patients with liver metastases compared with those without liver metastases (AST 34 vs 20 U/L; p < 0.001) (ALT 33 vs 21 U/L; p < 0.001) (ALP 123 vs 89 U/L; p < 0.001) (TB 0.5 vs 0.4 mg/dl; p = 0.036). Most patients with liver metastases had elevated AST (51% vs 13%; p < 0.001) or ALP levels (55% vs 23%; p < 0.001) above the ULN. ALT was elevated above the ULN in only 30% and TB in 10% of patients with liver metastases but the difference was still significant compared with controls (ALT 9%; p = 0.001) (TB 1%; p = 0.029). Patients with liver metastases presented with AST, ALT, or ALP ≥3 × upper limit of normal (ULN) in 27%, 13% and 33% of patients. However, none of the patients without liver metastases had liver test elevations ≥3 × ULN, including those patients with bone metastases (Table 1).

#### Breast cancer

Median AST, ALT, ALP, and TB were also slightly (but significantly) higher in patients with liver metastases compared with those without liver metastases (AST 41 vs 19 U/L; p < 0.001) (ALT 30 vs 21 U/L; p < 0.001) (ALP 123 vs 78 U/L; p < 0.001) (TB 0.4 vs 0.4 mg/dl; p = 0.022). Most patients with liver metastases had elevated AST (60% vs 14%; p < 0.001) or ALP levels (56% vs 22%; p < 0.001) above the ULN. ALT was elevated above the ULN in only 26% and TB in 14% of patients with liver metastases but the difference was still significant compared with controls (ALT 5%; p = 0.002) (TB 4%; p = 0.048). Patients with liver metastases presented with AST, ALT, or ALP ≥3 × ULN in 43%, 39% and 21% of patients. However, none of the patients without liver metastases had liver test elevations ≥3 × ULN, including those patients with bone metastases (Table 2).

#### Colorectal cancer

Median liver enzyme levels were slightly (but significantly) higher in patients with liver metastases compared with those without liver metastases (AST 36 vs 21 U/L; p < 0.001) (ALT 26 vs 24 U/L; p = 0.018) (ALP 135 vs 94 U/L; p < 0.001) but not for TB (0.5 vs 0.4 mg/dl; p > 0.05). Most patients with liver metastases had elevated AST (52% vs 22%; p = 0.001) or ALP levels (56% vs 24%; p < 0.001) above the ULN. ALT was elevated above the ULN in only 29% and TB in 15% of patients with liver metastases but the difference was still significant compared with controls (ALT 6%; p = 0.001) (TB 4%; p = 0.043). Patients with liver metastases presented with AST, ALT, or ALP ≥3 × ULN in 37%, 25% and 29% of patients. However, none of the patients without liver metastases had liver test elevations ≥3 × ULN, including those patients with bone metastases (Table 3).

#### Melanoma

Median AST and ALP were slightly (but significantly) higher in patients with liver metastases (46 vs 28 U/L; p = 0.006) (161 vs 96 U/L; p < 0.001) compared with those without liver metastases. However, while ALT was also slightly higher, the difference did not reach statistical significance (49 vs 33 U/L; p > 0.05). Values for TB were not different between the groups (0.6 vs 0.6 mg/dl; p > 0.05). Most patients with liver metastases had elevated AST or ALP levels (71%; p = 0.003) above the ULN. ALT was elevated above the ULN in only 47% and TB in 24% of patients with liver metastases but the difference was still significant compared with controls (ALT 13%); p = 0.031; (TB 0%; p = 0.038). Patients with liver metastases presented with AST, ALT, or ALP ≥3 × ULN in 33% of cases. However, none of the patients without liver metastases had liver test elevations ≥2 × ULN (Table 4).

### Liver biochemical values as screening tests for liver metastases

#### Lung cancer

The sensitivities and specificities of elevated liver tests above the ULN for the presence of liver metastases were as follows: AST 51%, 87%; ALT 30%, 91%; ALP 55%, 77%. The optimal cutoff for diagnosis of liver metastases according to sensitivity and specificity was ≥1.5 × ULN for AST (73%, 63%), ≥1.5 × ULN for ALT (50%, 83%), and >ULN for ALP (55%, 76%) (Table 5).

**Table 5.** Screening tests for liver metastases.

Liver enzyme elevation	Sensitivity (%)	Specificity (%)	PV+	PV-	AUC
**Lung cancer**					
AST >ULN	51.25	86.96	0.82	0.61	0.69
ALT >ULN	30.00	91.30	0.80	0.53	0.61
ALP >ULN	55.00	76.81	0.73	0.60	0.66
AST ≥1.5 × ULN	73.17	62.50	–	–	0.74
ALT ≥1.5 × ULN	50.00	83.33	–	–	0.72
ALP ≥1.5 × ULN	53.49	66.67	–	–	0.66
**Breast cancer**					
AST >ULN	60.00	86.21	0.79	0.71	0.73
ALT >ULN	26.00	94.00	0.81	0.60	0.60
ALP >ULN	56.00	77.59	0.68	0.67	0.67
AST ≥1.5 × ULN	76.67	75.00	–	–	0.80
ALT ≥1.25 × ULN	76.92	100.00	–	–	0.88
ALP ≥1.5 × ULN	64.29	76.92	–	–	0.73
**Colon cancer**					
AST >ULN	51.47	77.78	0.74	0.56	0.65
ALT >ULN	29.41	94.44	0.87	0.52	0.62
ALP >ULN	55.88	75.93	0.75	0.58	0.66
AST ≥2 × ULN	60.00	90.00	–	–	0.81
ALT ≥1.25 × ULN	85.00	33.33	–	–	0.57
ALP ≥1.5 × ULN	78.95	84.62	–	–	0.86
**Melanoma**					
AST >ULN	70.59	81.25	80.00	0.72	0.76
ALT >ULN	47.06	87.50	80.00	0.61	0.67
ALP >ULN	70.59	81.25	80.00	0.72	0.76
AST ≥1.5 × ULN	66.67	100.00	–	–	0.86
ALT ≥1.25 × ULN	66.67	50.00	–	–	0.67
ALP ≥1.5 × ULN	66.67	100.00	–	–	0.86

ULN range for each liver test: AST >33, ALT >49 and ALP >117.

AUC: Area under the curve; PV+: Positive predictive value; PV-: Negative predictive value; ULN: Upper limit of normal.

#### Breast cancer

The sensitivities and specificities of elevated liver tests above the ULN for the presence of liver metastases were as follows: AST 60%, 86%; ALT 26%, 95%; ALP 56%, 78%. The optimal cutoff for diagnosis of liver metastases according to sensitivity and specificity was ≥1.5 × ULN for AST (77%, 75%) and ALP (77%, 100%), and ≥1.25 × ULN for ALT (64%, 77%) (Table 5).

#### Colorectal cancer

The sensitivities and specificities of elevated liver tests above the ULN for the presence of liver metastases were as follows: AST 51%, 78%; ALT 29%, 94%; ALP 56%, 76%. The optimal cutoff for diagnosis of liver metastases according to sensitivity and specificity was ≥2 × ULN for AST (60%, 90%), ≥1.25 × ULN ALT (85%, 33%), and ≥1.5 × ULN for ALP (79%, 85%) (Table 5).

#### Melanoma

The sensitivities and specificities of elevated liver tests above the ULN for the presence of liver metastases were as follows: AST 71%, 81%; ALT 47%, 88%; ALP 71%, 81%. The optimal cutoff for diagnosis of liver metastases according to sensitivity and specificity was ≥1.5 × ULN for AST (68%, 100%) and ALP (68%, 100%), and ≥1.25 × ULN for ALT (68%, 50%) (Table 5).

## Discussion

Metastatic disease from various primary malignancies frequently involves the liver. It has been hypothesized that localization of metastases to the liver may be related to its dual blood supply resulting in increased exposure to circulating tumor cells from cancers originating from the gastrointestinal tract [2]. Other malignancies such as melanoma and breast cancer are thought to have selective targets for liver cells [1]. The lung is another a primary site that tends to localize to the liver especially in the case of small cell lung cancer [3]. One large population-based study of patients with liver metastases found carcinomas to be the predominant etiology, which was followed by melanoma. Adenocarcinoma was the most common subtype identified among those with liver metastases, the majority being colorectal in origin [28]. In the present study, the primary malignancies with the highest rates of liver metastases were colorectal cancer (13.6%) and melanoma (13.1%). Simultaneous metastatic disease to the bones was more often present in patients with liver metastases than those without liver metastases, except for in patients with colorectal cancer. While patients with colorectal cancer had a low rate of bone metastases, the difference was not significant between those with and without liver metastases (7.4% vs 1.9%; p > 0.05). As ALP represents a group of enzymes derived predominately from liver and/or bone, our results suggest that it may serve as a better indicator of liver metastases when elevated in patients with colorectal cancer, a conclusion also reached by others [23].

The early diagnosis of liver metastases remains challenging as most patients are without symptoms to suggest involvement of the liver at the time of detection [3]. Our study demonstrated that nearly 80% of patients with lung or breast cancers presented without symptoms indicative of liver involvement, as was the case in approximately 30–50% of patients with melanoma or colorectal cancer. Liver metastases are often discovered incidentally or during interval surveillance of the primary malignancy resulting in a delayed diagnosis with limited opportunity for therapeutic intervention [3]. The advent of novel systemic and biologic therapies has reignited the interest in laboratory testing as a readily available and cost-efficient strategy for the early detection of liver metastases [3,8,13]. Although rising tumor markers may signal patients in need of further evaluation, they tend to be specific to the primary tumors themselves and lack the ability to differentiate between local versus distant progression or to the site of metastases [21]. Given the potential for effective treatment with timely diagnosis, the reassessment of the utility of biochemical liver tests in predicting the presence of liver metastases is warranted.

Some researchers have postulated that liver test elevations may be related to the increased synthesis and release of liver enzymes in the setting of cellular injury from the infiltrative burden of metastases [22,23]. Tumor cells may compete with hepatocytes for oxygen and nutrients or invade hepatic vessels resulting in ischemia [29]. There are cases reported in the literature of liver metastases mimicking cirrhosis upon presentation. Vivas *et al.* described a patient with malignant melanoma who presented with abdominal distention and ultrasound showing a heteroechogenic liver, ascites, and portal vein enlargement. AST and ALT were found to be 6 × ULN and a liver biopsy was performed with histopathology revealing diffuse melanoma infiltration with few hepatocytes [30]. Another article reported a patient with no significant past medical history who was found to have elevated liver tests, AST 491 U/L, ALT 450 U/L, ALP 235 U/L, and TB 1.3 mg/dl, and a CT scan showing a heterogenous-appearing liver with small ascites. Given progressive liver test elevations and development of coagulopathy, the patient underwent liver biopsy with histopathology demonstrating primary breast carcinoma metastases [29].

While several studies have investigated how individual liver test abnormalities correlate with the presence of liver metastases from specific primary cancer types, none have been definitive. Wu *et al.* examined biochemical liver markers in patients with colorectal liver metastases. The authors found the median AST and ALT to be significantly higher in patients with liver metastases than those without liver metastases; however, there was no significant difference in ALP and TB [8]. A study by Cao *et al.* determined that the median AST, ALT, and ALP were significantly greater in patients with breast cancer liver metastases compared with those without liver metastases, but not TB [13]. In the present study, median AST, ALT, ALP, and TB were significantly higher in patients with liver metastases compared with patients without liver metastases for breast and lung cancers. Patients with colorectal liver metastases had median AST, ALT and ALP that were significantly greater than in patients without liver metastases but not TB. While patients with melanoma liver metastases had higher median AST and ALP, there was no significant difference in median ALT or TB compared with patients without liver metastases.

A study by Saif *et al.* looked at the number of patients with elevations in ALP among patients with and without liver metastases from colorectal cancer. They found that a significantly higher percentage of patients with liver metastases had elevated ALP above the ULN than in those without liver metastases (74% vs 33%) [23]. In our study, the percentage of patients with elevated AST, ALT, ALP, or TB was significantly greater in patients with liver metastases when compared with patients without liver metastases for all four primary cancers. In addition, nearly 50% or more patients with liver metastases from each primary malignancy had an elevated AST or ALP at the time of diagnosis. AST, ALT, or ALP ≥3 × ULN were found in patients with liver metastases from all four cancer types. In breast, lung and colorectal cancers, patients without liver metastases did not have elevations of liver enzymes to this level including patients with bone metastases. In the case of melanoma, none of the patients without liver metastases had liver enzyme elevations ≥2 × ULN. Our data suggest that liver test elevations are common in patients with liver metastases and may have risen to high levels by the time the diagnosis is made with imaging.

In the present study, screening tests were performed to determine levels of liver enzymes with the highest sensitivity and specificity for liver metastases from each primary cancer type. The highest optimal cutoff for AST according to sensitivity and specificity for the presence of liver metastases was ≥1.5 × ULN for melanoma, lung, and breast cancers and ≥2 × ULN for colorectal cancer. For ALT, the optimal cutoff was ≥1.25 × ULN for melanoma, breast, and colorectal cancers and ≥1.5 × ULN for lung cancer. For ALP, the optimal cutoff was ≥1.5 × ULN for melanoma, breast, and colorectal cancers, and above the ULN for lung cancer. Our findings suggest that using these defined cutoff points above the ULN that correspond to the origin of the primary cancer may improve the diagnostic value of liver tests for liver metastases.

A major strength of this study was the patient population, which included a large number of cases with liver metastases from encounters at a National Cancer Institute-designated Comprehensive Cancer Center who were matched to controls by primary cancer type, age, and gender. Each ICD cancer diagnosis was confirmed by tissue biopsy from the primary site and each ICD diagnosis of liver metastases was confirmed by imaging and/or biopsy results documented EMR. Furthermore, patients with a prior history of liver disease or cirrhosis were excluded from the study to minimize confounding of pre-existing liver test elevations. The type of cancer treatment received did not significantly differ between patients with or without liver metastases for each primary malignancy except, in the case of lung cancer. However, most patients with lung cancer liver metastases had not yet received treatment of their primary malignancy. In colorectal cancer, there were few patients with metastatic disease to the bones, and the rates did not significantly differ between those with and without liver metastases. This suggests that the significant elevations in ALP observed in colorectal cancer patients with liver metastases compared with those without liver metastases may be attributed to the presence of liver rather than bone metastases.

Our study was limited by the retrospective observational study design. In addition, patient encounters were selected from an academic tertiary care center, and thus, our results may be subject to referral bias. Liver tests were obtained from the time of diagnosis of liver metastases on imaging and/or biopsy, and no inferences can be made about test results preceding or following the diagnosis of metastases. Nevertheless, our results are based on a large number of patients with documented liver metastases with adequate controls and we believe the positive predictive values of the liver tests will prove to be a valuable finding worthy of prompting additional evaluation in patients with these (and perhaps) other malignancies.

## Conclusion

We have demonstrated that liver tests are commonly elevated in the presence of liver metastases and may have reached high levels by the time they are detected on imaging. Our results suggest that using thresholds of liver enzymes above the standard laboratory ULN may improve the diagnostic accuracy for liver metastases and indicate which patients should undergo further evaluation with diagnostic imaging. These biochemical liver tests have the potential to serve as a rapid and cost-effective tool to use during surveillance of primary malignancies to predict the presence of liver metastases, even in the absence of symptoms. Longitudinal studies of liver tests following diagnosis of the primary cancer are needed to confirm that elevations are indicative of liver metastases prior to the detection with imaging.

**Summary points**

- Metastases to the liver generally portend unfavorable outcomes if left untreated; therefore, early detection is essential for improving patient prognosis.
- Although routinely performed as part of the workup, the utility of biochemical liver tests in the detection of liver metastases from common primary cancers localizing to the liver remains unclear.
- This was a retrospective analysis of biochemical liver tests in patients with malignant melanoma, breast, colorectal, or lung cancers at the time of diagnosis of liver metastases.
- In the present study, liver test elevations were found to be common among patients with liver metastases. The percentage of patients with elevated AST, ALT, ALP, or TB was significantly greater in patients with liver metastases when compared with patients without liver metastases for all four primary cancers.
- Liver tests in some cases had already reached high levels at the time of detection with imaging. AST, ALT, or ALP ≥3 × ULN were found among patients with liver metastases in each of the four cancer types.
- Using defined cutoff points for liver tests above the ULN according to the primary cancer type may increase the diagnostic value for the presence of liver metastases.
- The results of this study suggest that biochemical liver tests may serve as a rapid and cost-effective tool to use during surveillance of primary malignancies to predict the presence of liver metastases even in the absence of symptoms, and signal patients in need of further evaluation with diagnostic imaging.
- Future prospective studies are indicated to evaluate the potential for liver tests to detect liver metastases prior to diagnosis on imaging.
